# First cytogenetic information for *Lonchothrix emiliae* and taxonomic implications for the genus taxa *Lonchothrix* + *Mesomys* (Rodentia, Echimyidae, Eumysopinae)

**DOI:** 10.1371/journal.pone.0215239

**Published:** 2019-04-16

**Authors:** Leony Dias de Oliveira, Willam Oliveira da Silva, Marlyson Jeremias Rodrigues da Costa, Iracilda Sampaio, Julio Cesar Pieczarka, Cleusa Yoshiko Nagamachi

**Affiliations:** 1 Centro de Estudos Avançados da Biodiversidade, Laboratório de Citogenética, Instituto de Ciências Biológicas, Universidade Federal do Pará, Belém, Pará, Brazil; 2 Laboratório de Genética e Biologia Molecular, Universidade Federal do Pará, Campus Universitário de Bragança, Bragança, Pará, Brazil; Sichuan University, CHINA

## Abstract

The taxonomic identification of *Lonchothrix emiliae* (Rodentia, Echimyidae, Eumysopinae) is problematic because of the overlap of morphological characters with its sister clade represented by species in the genus *Mesomys* which, like *L*. *emiliae*, is distributed throughout the Amazonian biome. Cytogenetic studies reported the karyotype of *L*. *emiliae* as 2n = 60/FN = 116, but this karyotype and samples were later designated as *M*. *hispidus*. To evaluate the karyotype diversity of *Lonchothrix* and *Mesomys*, and to provide data useful as karyological diagnostic characters, in the present study we made a comparative analysis of specimens of *L*. *emiliae* and *M*. *stimulax* collected from two Brazilian Amazonian localities, using C-banding, G-banding, FISH using rDNA 45S and telomeric probes, and Cytochrome-b (Cytb) sequences. The results indicate that *L*. *emiliae* has 2n = 64♀, 65♂/FN = 124 and a multiple sexual system (XX/XY_1_Y_2_), while *M*. *stimulax* has 2n = 60/FN = 116. The Neo-X system found in *L*. *emiliae* also occurs in two *Proechimys* species, but cytogenetic analysis indicated an independent origin for these systems. The rDNA 45S analysis showed interstitial signals at one autosomal pair for each species, while an ITS found in *L*. *emiliae* was not coincident with the NOR. The molecular analysis confirmed *Lonchothrix* and *Mesomys* are sister genera, and the high level of intraspecific genetic divergence (7.1%) in *M*. *stimulax* suggests that it may be a species complex.

## Introduction

Echimyidae is the most diverse family of Hystricognathi rodents in South America, with 22 genera and 88 currently recognized species [1]. Some members of this family are arboreal and sub-sampled, taking into account that the most employed methods favor the capture of specimens with terrestrial and/or scansorial habits [2]. There are also difficulties in the taxonomic identification, as a result of overlap of morphological characters among distinct genera [1]. The monophyly of the nine genera included in the Eumysopinae subfamily has been questioned based on morphological and molecular analyses, in which polytomies with the Dactylomyinae and Echimyinae subfamilies have been observed [1, 3, 4]. However, *Lonchothrix* and *Mesomys* have been consistently recognized as sister taxa [1, 5].

**Fig 1:**
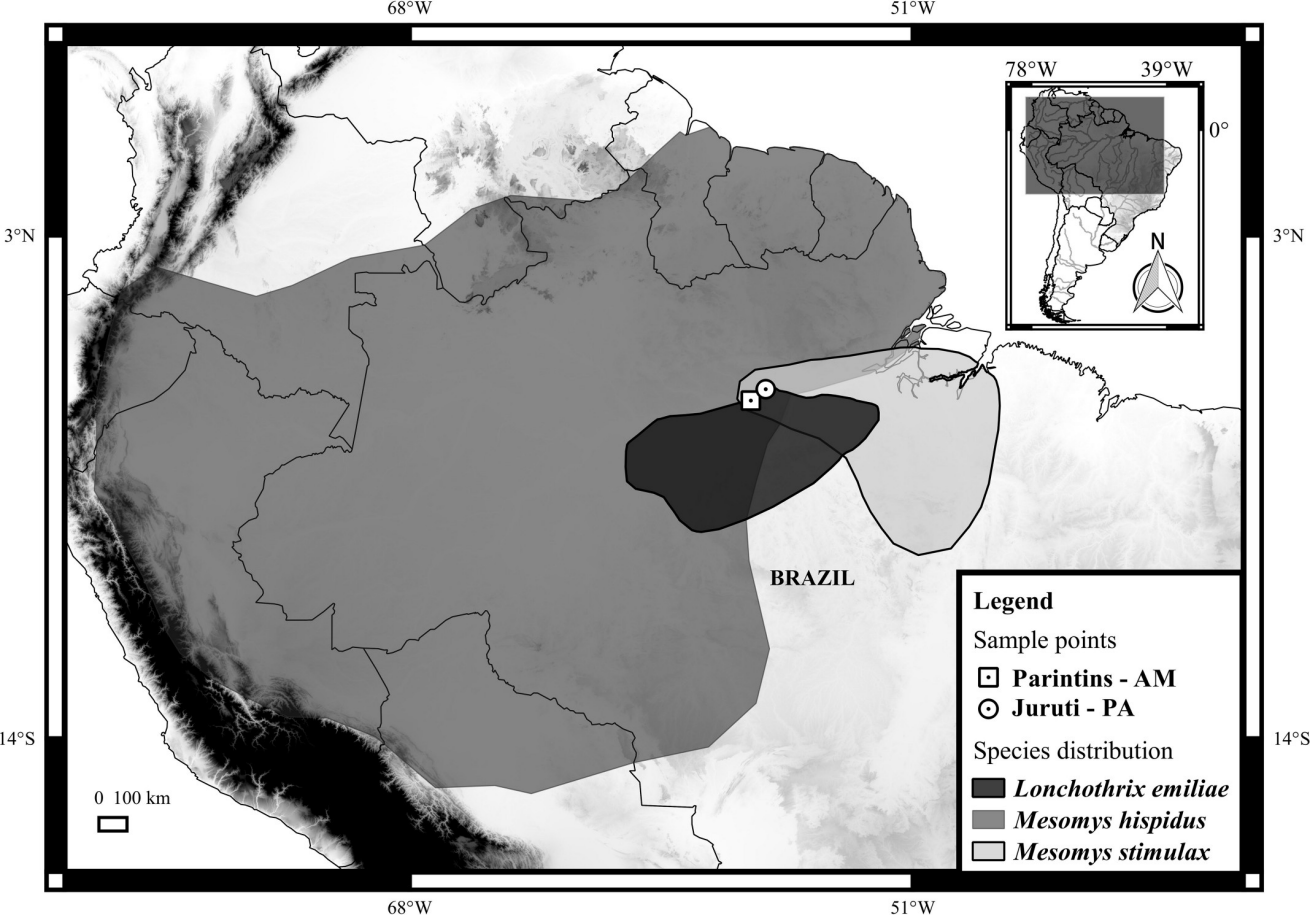
Geographic distribution of *Lonchothrix emiliae*, *Mesomys hispidus*, and *M*. *stimulax*, and the sampling sites for *L*. *emiliae* and *M*. *stimulax*. **Parintins–AM (white square with black dot), Juruti–PA (white circle with black dot).** The Brazilian states are Amazonas (AM) and Pará (PA). The map was generated using QUANTUM-GIS software, version 3.6. Databases were obtained from DIVA-GIS [16], and IUCN (International Union for Conservation of Nature) [17–19].

*Lonchothrix* is a monotypic genus (*L*. *emiliae*) and endemic to the Brazilian Amazon, from the lower Madeira, Tapajós, and Xingu Rivers to south of the Amazonas River (northern Brazil), and is sympatric with two *Mesomys* species (*M*. *hispidus* and *M*. *stimulax*) (Fig 1). On its part, *Mesomys* as genus contains four valid species (*M*. *hispidus*, *M*. *leniceps*, *M*. *occultus*, and *M*. *stimulax*), and is distributed across the Amazon Basin and Guyana regions, from the eastern Andes to the central part of Brazil and northern Bolivia [1].

Cytogenetic data for *L*. *emiliae* samples collected in Peru show 2n = 60/FN = 116 [6]. However, Patton et al. [7] associated this karyotype to samples of *M*. *hispidus* collected in several Brazilian Amazon localities, and suggested that there was a mistake in the taxonomic identification made by Aniskin [6]. Consequently, there would have no cytogenetic data available for *Lonchothrix* [8] (Table 1).

**Table 1:** Cytogenetic data for the *Lonchothrix* and *Mesomys* genera, including the diploid number (2n), the autosomal fundamental number (FN), the locality, and appropriate references.

Species	2n	FN	Locality	Reference
*L*. *emiliae*	64♀/65♂	124	Juruti, Pará, Brazil (02°11’54.6”S 55°58’15”W), Parintins, Amazonas, Brazil (02°34’45.7”S 56°28’14.4”W)	Present study
*M*. *stimulax*	60	116	Juruti, Amazonas, Brazil (2°34’45.7”S 56°28’14.4”W)	Present study
*M*. *hispidus*	60	116	Madeira and Juruá Rivers, Amazonas, Brazil (06°35’S 68°54’W)	[7, 9]
*M*. *occultus*	42	54	Rio Juruá, Amazonas, Brazil (3°17’S 66°14’W)	[7, 9]
*M*. *stimulax*	60	110, 116	Altamira, Pará, Brazil (6°38’S 69°52’W); Parauapebas, Pará, Brazil (05°6’12”S 49°53’18”W)	[7, 8]

Based on conventional staining, two karyotypes have been reported for *M*. *stimulax* (2n = 60/FN = 116 and FN = 110) [1, 8] and one for *M*. *occultus* (2n = 42/FN = 54) [7, 9] (Table 1).

To evaluate the karyotypic diversity of *Lonchothrix* and *Mesomys* and provide data on a potential taxonomic marker, we analyzed and compared specimens of *L*. *emiliae* and *M*. *stimulax* collected in two Brazilian Amazon localities, using classic and molecular cytogenetics and Cytochrome-b (Cytb) sequences. We discuss the chromosomal diversity found and its implications for distinguishing these taxa taxonomically.

## Materials and methods

### Samples

The *Lonchothrix emiliae* specimens included two males from Juruti (Pará state) and one male and one female from Parintins (Amazonas state), and the *Mesomys stimulax* specimens comprised four males collected from Juruti (Fig 1, Table 1). The specimens were collected using pitfall traps [10], and have been deposited in the mammal collection of the Museu de Zoologia da Universidade Federal do Pará (UFPA; Belém, Pará, Brazil). The animals collected were handled according to American Society of Mammalogists procedures. The rodents were maintained in the lab with food and water, free from stress, until their euthanasia using intraperitoneal injection of barbiturate (Pentobarbital, 120 mg/kg) after local anesthetic (lidocaine used topically). JCP has a permanent field permit, number 13248 from “Instituto Chico Mendes de Conservação da Biodiversidade”. The Cytogenetics Laboratory from UFPa has permit number 19/2003 from the Ministry of Environment for sample transport and permit 52/2003 for using the samples for research. The Ethics Committee (Comitê de Ética Animal da Universidade Federal do Pará) approved this research (Permit 68/2015).

### Cytogenetic analysis

Chromosomal preparations were obtained from bone marrow [11], and C-banding [12], G-banding [13], and FISH (Fluorescence *In Situ* Hybridization) using telomeric (ONCOR) and rDNA 45S probes [14] were performed. All techniques were adapted from the original protocols. Classic cytogenetic images were obtained using an Olympus BX41 microscope (bright field/phase) with a digital camera CCD 1300QDS, and analyzed using SpectraView software (Applied Spectral Imaging). FISH images were obtained using a Nikon H550S microscope, and analyzed using Nis-Elements software. Chromosomes morphology was classified according to Levan et al. [15] with modifications.

The map was made using QUANTUM-GIS (QGIS) program version 3.6 Database were obtained from DIVA [16] and IUCN (International Union for Conservation of Nature) [17–19].

### Molecular analysis

We used 803 base pair (bp) sequences of the Cytochrome b mitochondrial gene (Cytb) from 26 samples. These included 5 sequences obtained from new samples (one from *M*. *stimulax* and four from *L*. *emiliae*), and 21 sequences obtained from GenBank (S1 Table). *Octodon degus* was used as outgroup, as it belongs to the Octodontidae family and is phylogenetically close to the Echimyidae [20].

DNA extractions from muscular tissue were made using the QIAamp DNA Mini Kit (Qiagen), according to the manufacturer's protocol. We used the primers ‘MVZ 05’ and ‘MVZ 16’ [21] to amplify Cytb gene fragments in a 96-well Veriti thermal cycler (Applied Biosystems), and these were sequenced in an automatic sequencer (Genetic Analyzer 3500 XL, Applied Biosystems) using only the primer ‘MVZ 05’. The alignment and editing were conducted using the BioEdit Sequence Alignment Editor program, version 7.0.5.2 [22]. A search for the best nucleotide substitution model was made using jModeltest 2.0.2 software [23]. Base saturation was tested using DAMBE5 software [24]. The maximum likelihood (ML) analysis was based on 1,000 bootstrap replicates using PhyML software [25, 26]. The phylogenetic tree was edited using the Figtree program, version 1.4.2 [27].

## Results

### Classic and molecular cytogenetics

**Fig 2:**
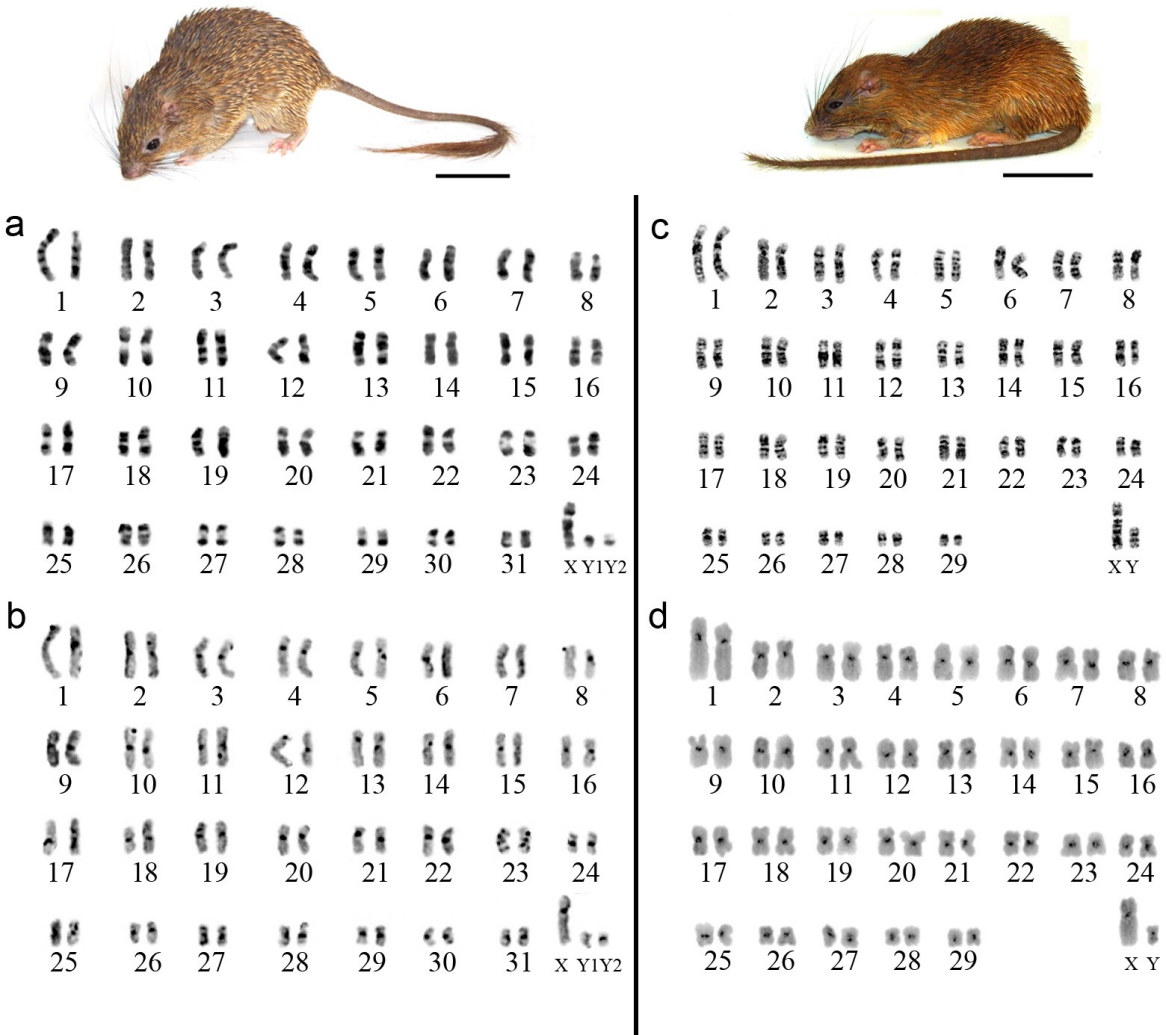
**G-banding (a, c) and C-banding (b, d) patterns for the *Lonchothrix emiliae* (a, b; 2n = 64♀, 65♂/FN = 124) and *Mesomys stimulax* (c, d; 2n = 60/FN = 116) karyotypes**. The images of the respective species analyzed are shown above the karyotypes: *L*. *emiliae* (left) and *M*. *stimulax* (right). Scale bar: 10 cm.

The karyotype of *L*. *emiliae* shown 2n = 64♀, 65♂/FN = 124, with a multiple sexual chromosome system (XX/XY_1_Y_2_), and all autosomes had meta/submetacentric morphology and ranged from large to small. The X chromosome was a mid-sized submetacentric, the Y_1_ chromosome was a small acrocentric, and the Y_2_ chromosome was a small submetacentric (Fig 2A). Constitutive heterochromatin (CH) was distributed in the pericentromeric region of all autosomes, including the X chromosome; in the Y_1_ and Y_2_ chromosomes it was in the centromeric region (Fig 2B). The karyotype of *M*. *stimulax* shown 2n = 60/FN = 116, and all autosomes had meta/submetacentric morphology that ranged in size from large to small. The X chromosome was a mid-sized submetacentric, and the Y chromosome was a small metacentric (Fig 2C). The CH was distributed in the pericentromeric region of all chromosomes, including the X and Y (Fig 2D).

**Fig 3:**
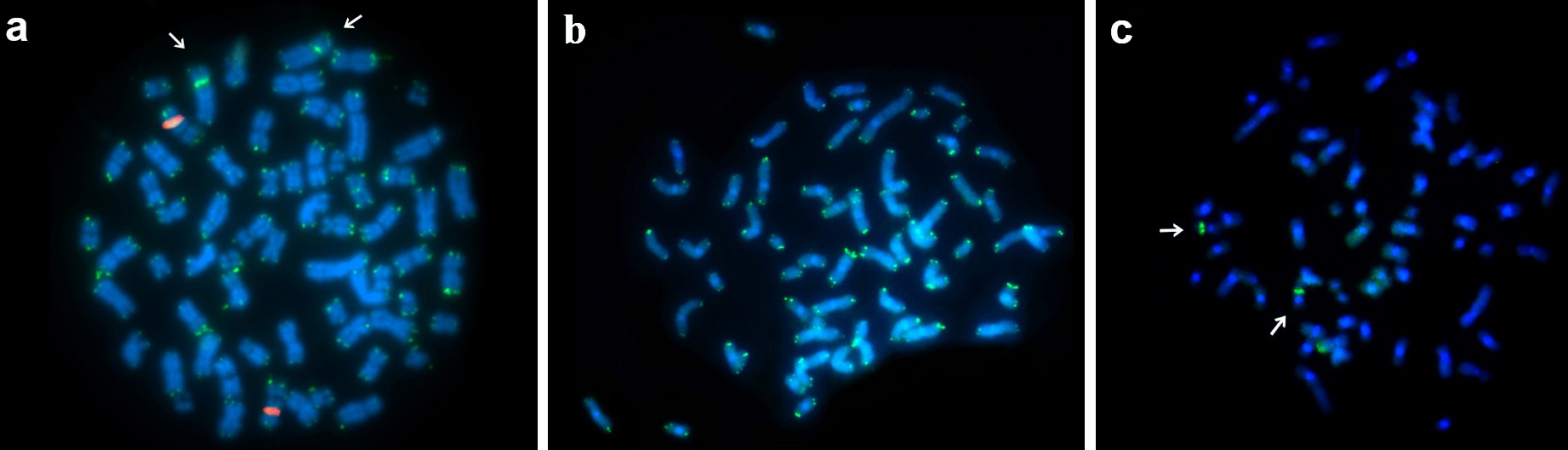
**FISH using rDNA 45S (a, c) and telomeric (a, b) probes for *L*. *emiliae* (a) and *M*. *stimulax* (b, c).** Arrows indicate the ITS for *L*. *emiliae* metaphase (a), and rDNA 45S for *M*. *stimulax* metaphase (c).

For both *L*. *emiliae* (Fig 3A) and *M*. *stimulax* (Fig 3C) the FISH analysis using rDNA 45S probes showed that the NOR was in the interstitial region of a single autosomal pair. FISH using telomeric probes showed hybridization at the distal region of all chromosomes in both species (Fig 3A and 3B). An interstitial telomeric sequence (ITS) was observed in the short arm of pair 4 of *L*. *emiliae* (Fig 3A).

### Molecular phylogeny

**Fig 4:**
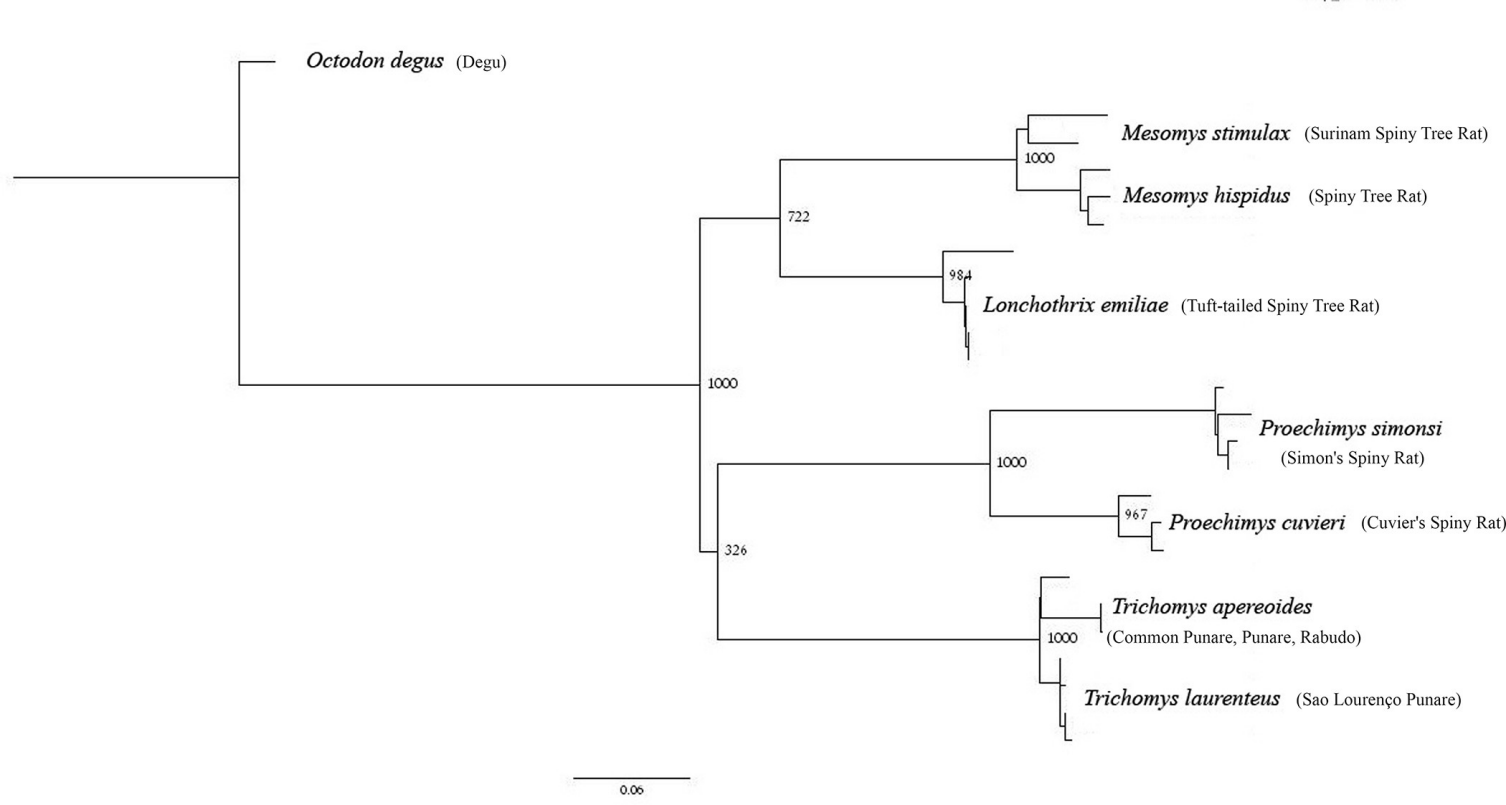
Maximum likelihood tree for seven Echimyidae species obtained using PhyML, based on the mitochondrial gene Cyt b (803 bp). The numbers near the nodes are bootstrap values for 1,000 replicates. Species common names are within the parenthesis.

All analyzed species of the Echimyidae family grouped with 100% bootstrap support in the maximum likelihood analysis (Fig 4). The *Lonchothrix* + *Mesomys* relationship had medium support (72%), while the support was 100% for each of the intrageneric groupings *M*. *hispidus* + *M*. *stimulax*, *Proechimys simonsi* + *P*. *cuvieri*, and *Trichomys apereoides* + *T*. *laurenteus*.

We found intergeneric divergence of 18 ± 28% among the taxa analyzed (Table 2). The intrageneric distance was smaller for the *T*. *apereoides* + *T*. *laurenteus* group (4%), it was 9% for the *M*. *hispidus* + *M*. *stimulax* group, and the *P*. *simonsi* + *P*. *cuvieri* group had the greatest intrageneric distance (18%). The intraspecific medium nucleotide divergence for each species was < 3.5%, except for *M*. *stimulax* (7.1%) (Table 2).

**Table 2:** *P*-distance estimates for the Cytochrome b (*Cytb*) gene among the monophyletic clades recovered in the phylogenetic analysis. IV = intraspecific variation. Bold scores represent the lowest and highest values of interspecific and intraspecific divergence for the different species analyzed.

**Species**	**1**	**2**	**3**	**4**	**5**	**6**	**7**	**IV(%)**
*Mesomys stimulax*								**7.1**
*Mesomys hispidus*	0,09							2.6
*Lonchothrix emiliae*	0,22	**0,18**						2.1
*Trichomys apereoides*	0,23	0,24	0,23					3.2
*Trichomys laurenteus*	0,23	0,23	0,23	**0,04**				**0.4**
*Proechimys simonsi*	0,23	0,26	0,25	0,28	0,28			2.8
*Proechimys cuvieri*	0,26	0,26	0,24	0,26	0,27	**0,18**		1.6
*Octodon degus*	0,27	0,26	0,22	0,28	0,28	0,28	0,27	1.8

## Discussion

### Taxonomic implications and karyotype analysis

*Mesomys* and *Lonchothrix* have been considered in various studies as sister genera, based on morphological and ecological similarities [1, 28], and a high support as a monophyletic clade based on molecular analyses [4, 28, 29]. Some authors even suggest that they should form a single genus [1, 28]. In addition, taxonomic evaluation of *Lonchothrix* and *Mesomys* using caudal morphology as a diagnostic character is problematic because of autotomy in these taxa [30]. Other studies have demonstrated a high intraspecific genetic divergence among clades of *M*. *hispidus*, which leads inconclusive the specific status of this taxon [9].

The cytogenetic data from the present study showed that the karyotype of *L*. *emiliae* (2n = 64♀, 65♂/FN = 124) is distinct from that of the *Mesomys* genus, in which 2n ranges from 42 to 60, and FN from 54 to 116 (Table 1). Thus, our findings corroborate those of Patton et al. [7], who proposed that the karyotype 2n = 60/FN = 116 described by Aniskin [6] supposedly for “*L*. *emiliae*” samples from Peru was in fact derived from *M*. *hispidus* samples, and that this resulted from incorrect taxonomic identification. Therefore, the chromosomal data provided in the present study are the first ones for *Lonchothrix* and can be used as an auxiliary tool in taxonomic distinction of these genera.

The *M*. *stimulax* samples in the present study showed the same karyotype (2n = 60/FN = 116) reported by Patton et al. [7] for this taxon, which is distinct from the 2n = 60/FN = 110 karyotype reported by Nagamachi et al. [8] for samples collected in Parauapebas (southeast of Pará state, Brazil). Pericentric inversions could explain the difference between these two FN values. Our findings of chromosomal banding and from FISH using telomeric and ribosomal probes for this species could be used to enhance understanding of the karyotype evolution of the *Mesomys* genus.

The karyotypes of *Mesomys* and *Lonchothrix* each yielded rDNA 45S hybridization signals at one autosomal pair, in both cases in the interstitial region. However, the ITS (interstitial telomeric sequence) found on pair 4 of *L*. *emiliae* was not coincident with the NOR (Fig 3A). This ITS could be a trait resulting from a chromosomal rearrangement such as fusion [31]. This has been reported for other Echimyidae taxa including *Proechimys goeldii*, in which the ITS in the centromeric region was associated with autosome/sexual fusion [32]. Alternatively, the ITS could be the result of accumulation of heterochromatin containing the telomeric sequence.

The presence of multiple sex determination systems (XY_1_Y_2_) has been described for other members of the Echimyidae family including *Proechimys* cf. *longicaudatus* (2n = 14♀, 15♂/16♀, 17♂; Amaral et al. [33]) and *P*. *goeldii* (2n = 24♀, 25♂/26♀, 27♂; Rodrigues da Costa et al. [32]). Based on chromosomal morphology and size, meiotic behavior, and comparison of G-banding patterns, Rodrigues da Costa et al. [32] proposed that the Neo-X chromosomes in *Proechimys* species are a homoplasic characteristic and not evidence of a common ancestry. Based on comparison of the *Proechimys* and *Lonchothrix* Neo-X chromosomes, we hypothesize that this chromosome originated independently in each of these three taxa.

### Molecular analysis of *Lonchothrix* and *Mesomys*

Molecular analysis showed that all species we analyzed from the Echimyidae family (*L*. *emiliae*, *M*. *hispidus*, *M*. *stimulax*, *P*. *cuvieri*, *P*. *simonsi*, *T*. *apereoides*, and *T*. *laurenteus*) (Fig 4) are monophyletic, confirming the taxonomic identification of our samples (*L*. *emiliae* and *M*. *stimulax*) with their respective sequences from GenBank. However, their sister genera relationship had moderate support from the bootstrap analysis (72%). This result was expected as the Cytb gene has better resolution at terminal branches than basally, as described for other taxa in the Echimyidae family [7, 20, 28, 29, 32, 34, 35].

The two *Mesomys* species (*M*. *hispidus* and *M*. *stimulax*) showed 9% interspecific genetic divergence. However, *M*. *hispidus* showed 2% intraspecific divergence while for *M*. *stimulax* it was 7%. As emphasized previously [36], the genus *Mesomys* has not been revised since Desmarest (1817) described the first species (see Patton et al. [1] for a general review of the nomenclatural history of this genus). Nevertheless, the molecular analyses [7, 9] identified a number of divergent and geographically structured clades within *M*. *hispidus* that may warrant species status when additional sampling and analyses are undertaken. We observed that both *M*. *hispidus* and *M*. *stimulax* have different levels of molecular divergence associated with geographic patterns, reinforcing the need for a more detailed revision of the species of this genus.

According to Bradley & Baker [37] and Baker & Bradley [38], who performed a meta-analysis of the Cytb gene from different rodent species, genetic divergence values > 5% are associated with potentially undescribed species. Recent studies in Amazonian rodents demonstrated that three new lineages of *Neacomys* (Cricetidae), with different karyotypes, show genetic divergence (Cytb and COI) ranging from 6.2 to 9.1% [39], leading the authors to propose that these lineages would be new species candidates. A similar result was found on two subpopulations of *Proechimys goeldii* (*Proechimys*, Echimyidae) with different karyotype and genetic divergence values >6% (Cytb) [32]. The authors questioned the taxonomic status of *P*. *goeldii* and suggested to be a case of cryptic species [32], in which distinct species lack discriminatory morphological characters [40]. We are dealing with a similar situation in the present work. As proposed to *Mesomys hispidus* [7, 9], we suggest that a taxonomic review is needed for a better understanding of *M*. *stimulax* taxonomic status, and indicates that it may constitute a species complex.

## Conclusions

Our study provides the first cytogenetic data for *Lonchothrix emiliae* (2n = 64♀, 65♂/FN = 124) that can contribute to accurate taxonomic identification of this taxon, which is often confused with *Mesomys*. Based on our molecular and karyotypic data, we reinforce that these taxonomic entities are two distinct genera. In addition, the molecular data for *Mesomys* indicates that a substantial taxonomic revision of this group is needed to clarify its geographic boundaries, number of species, and evolutionary history. The Neo-X chromosome in each of *L*. *emiliae* and *Proechimys* may have resulted from independent events within family Echimyidae, in which case these species may be useful models for studies of sex chromosome evolution.

## Supporting information

S1 Table List of specimens included in the molecular analysis of the Cytochrome b gene (Cytb) in the present study. For each sample the GenBank number/voucher, locality, and reference are provided.
(DOCX)
