# Supplementary material for: First cytogenetic information for Lonchothrix emiliae and taxonomic implications for the genus taxa Lonchothrix + Mesomys (Rodentia, Echimyidae, Eumysopinae)
Source: PLoS One. 2019 Apr 16;14(4):e0215239. doi: 10.1371/journal.pone.0215239 (PMC6467446; doi:10.1371/journal.pone.0215239)
Supplement: S1 Table — For each sample the GenBank number/voucher, locality, and reference are provided. (DOCX) [file pone.0215239.s001.docx]

**Supplementary Table 1.** List of specimens included in the molecular analysis of *Cytochrome b* (Cytb) in the present study. For each sample, the GenBank number/Voucher, locality and reference are provided.

| **Species** | **GenBank/**  **Voucher number** | **Locality** | **Reference** |
| --- | --- | --- | --- |
| *Lonchothrix emiliae* | AF422921.1 | Alter do Chão, Pará, Brazil | Leite & Patton (2002) |
|  | UFPAM2036 | Parintins, Amazonas, Brazil | Present study |
|  | UFPAM2037 | Parintins, Amazonas, Brazil | Present study |
|  | UFPAM1997 | Juruti, Pará, Brazil | Present study |
|  | UFPAM2012 | Juruti, Pará, Brazil | Present study |
| *Mesomys stimulax* | KU892788.1 | Nickerie, Surinam | Fabre et al. (2016) |
|  | LTJ65 | Juruti, Pará, Brazil | Present study |
| *Mesomys hispidus* | L23385.1 | Penedo, Amazonas, Brazil | da Silva & Patton (1993) |
|  | L23395.1 | Barro Vermelho, Amazonas, Brazil | da Silva & Patton (1993) |
|  | KF590696.1 | Not available | Upham et al. (2013) |
| *Proechimys simonsi* | EU313249.1 | Not available | Petterson & Velazco (2008) |
|  | EU313250.1 | Not available | Petterson & Velazco (2008) |
|  | EU35414.1 | Aguas Calientes, Peru | Petterson & Velazco (2008) |
| *Proechimys cuvieri* | AJ251400.1 | French Guiana | Steiner et al. (2000) |
|  | AJ251401.1 | French Guiana | Steiner et al. (2000) |
|  | AJ251402.1 | French Guiana | Steiner et al. (2000) |
|  | AJ251403.1 | French Guiana | Steiner et al. (2000) |
| *Trichomys apereoides* | U34854.1 | Santo Inácio, Bahia, Brazil | Lara et al. (1996) |
|  | AY083336.1 | Not available | Braggio & Bonvicino (2004) |
|  | AY083337.1 | Not available | Braggio & Bonvicino (2004) |
| *Trichomys laurenteus* | JX459856.1 | Coronel José Dias, Piauí, Brazil | Nascimento et al. (2013) |
|  | JX459857.1 | Coronel José Dias, Piauí, Brazil | Nascimento et al, (2013) |
|  | JX459858.1 | Coronel José Dias, Piauí, Brazil | Nascimento et al. (2013) |
|  | JX459859.1 | Coronel José Dias, Piauí, Brazil | Nascimento et al. (2013) |
| *Octodon degus* | AF422914.1 | Chile | Leite & Patton (2002) |
|  | AM407929.1 | Chile | Huchon et al. (2007) |

**References**

Braggio E, Bonvicino CR. Molecular Divergence in the Genus *Thrichomys* (Rodentia, Echimyidae), J Mammal. 2004; vol. 85, pp. 316-320.

Fabre PH, Upham NS, Emmons LH, Justy F, Leite YLR, Loss AC, et al. Mitogenomic Phylogeny, Diversification, and Biogeography of South American Spiny Rats. Mol Bio and Evol. 2016; 34: 613-633.

Huchon D, Chevret P, Jordan U, Kilpatrick CW, Ranwez V, Jenkins PD, et al. Multiple molecular evidences for a living mammalian fossil. PNAS. 2007; 104(18): 7495-9.

Lara MC, Patton JL, Silva MN. The simultaneous diversification of South American Echimyid rodents (Hystricognathi) based on complete cytochrome b sequences. Mol Phylogen Evol. 1996; 5: 403–413.

Leite YLR, Patton JL. Evolution of South American spiny rats (Rodentia, Echimyidae): the star-phylogeny hypothesis revisited. Mol Phylogen Evol. 2002; 25: 455–464.

Nascimento FF, Lazar A, Menezes AN, Durans AD, Moreira JC, Salazar-Bravo J, et al. The Role of Historical Barriers in the Diversification Processes in Open Vegetation Formations during the Miocene/Pliocene Using an Ancient Rodent Lineage as a Model. PloS One. 2013; 8(4): e61924.

Patterson BD, Velazco PM. Phylogeny of the Rodent Genus Isothrix (Hystricognathi, Echimyidae) and its Diversification in Amazonia and the Eastern Andes. J Mamm Evol*.* 2008; 15: 181-201.

Silva MN, Patton JL. Amazonian phylogeography: mtDNA sequence variation in arboreal echimyid rodents (Caviomorpha). Mol Phylog and Evol. 1993; 243-55.

Steiner M, Sourrouille P, Catzeflis F. Molecular characterization and mitochondrial sequence variation in two sympatric species of Proechimys (Rodentia: Echimyidae) in French Guiana. Biochem Syst and Ecol. 2000; 28(10): 963-973.

Upham, NS, Ojala-Barbour R, JorgeBrito M, Velazco PM, & Patterson BD. Transitions between Andean and Amazonian centers of endemism in the radiation of some arboreal rodents. BMC Evol Biol. 2013; 13: 191.
